# The association between exaggeration in health related science news and academic press releases: retrospective observational study

**DOI:** 10.1136/bmj.g7015

**Published:** 2014-12-10

**Authors:** Petroc Sumner, Solveiga Vivian-Griffiths, Jacky Boivin, Andy Williams, Christos A Venetis, Aimée Davies, Jack Ogden, Leanne Whelan, Bethan Hughes, Bethan Dalton, Fred Boy, Christopher D Chambers

**Affiliations:** 1 Cardiff University Brain Research Imaging Centre, School of Psychology, Cardiff University, Cardiff CF10 3AT, UK; 2 School of Psychology, Cardiff University, UK; 3 School of Journalism, Media & Cultural Studies, Cardiff University, UK; 4 School of Women’s and Children’s Health, University of New South Wales, and Graduate School of Medicine, Faculty of Science, Medicine and Health, University of Wollongong, Australia; 5 Department of Psychology, Swansea University, UK

## Abstract

**Objective** To identify the source (press releases or news) of distortions, exaggerations, or changes to the main conclusions drawn from research that could potentially influence a reader’s health related behaviour.

**Design** Retrospective quantitative content analysis.

**Setting** Journal articles, press releases, and related news, with accompanying simulations.

**Sample** Press releases (n=462) on biomedical and health related science issued by 20 leading UK universities in 2011, alongside their associated peer reviewed research papers and news stories (n=668).

**Main outcome measures** Advice to readers to change behaviour, causal statements drawn from correlational research, and inference to humans from animal research that went beyond those in the associated peer reviewed papers.

**Results** 40% (95% confidence interval 33% to 46%) of the press releases contained exaggerated advice, 33% (26% to 40%) contained exaggerated causal claims, and 36% (28% to 46%) contained exaggerated inference to humans from animal research. When press releases contained such exaggeration, 58% (95% confidence interval 48% to 68%), 81% (70% to 93%), and 86% (77% to 95%) of news stories, respectively, contained similar exaggeration, compared with exaggeration rates of 17% (10% to 24%), 18% (9% to 27%), and 10% (0% to 19%) in news when the press releases were not exaggerated. Odds ratios for each category of analysis were 6.5 (95% confidence interval 3.5 to 12), 20 (7.6 to 51), and 56 (15 to 211). At the same time, there was little evidence that exaggeration in press releases increased the uptake of news.

**Conclusions** Exaggeration in news is strongly associated with exaggeration in press releases. Improving the accuracy of academic press releases could represent a key opportunity for reducing misleading health related news.

## Introduction

The framing of health related information in the national and international media, and the way in which audiences decode it, has complex and potentially powerful impacts on healthcare utilisation and other health related behaviour in many countries.^1^ ^2^ ^3^ ^4^ ^5^ ^6^ The media also demonstrably influences the behaviour of scientists and doctors.^3^ ^4^ Such impacts may often be beneficial, but misleading messages can have adverse effects (even if these effects may be difficult to predict and prove because the responses of audiences are complex and multiply determined).^6^ This problem is not restricted to rare dramatic cases such as vaccination scares^7^ ^8^; the cumulative effect of everyday misreporting can confuse and erode public trust in science and medicine, with detrimental consequences.^9^ ^10^ ^11^

“Information subsidies” such as university press releases have long been used to deliver salient aspects of selected research,^12^ ^13^ and as journalists are increasingly expected to produce more copy in less time^14^ ^15^ these press releases have become the dominant link between academia and the media.^16^ ^17^ As such, information included in press releases is highly likely to be included in news stories.^18^ Although accurate information, alone, is not sufficient for clear public understanding and informed behaviour,^19^ it is nevertheless important that health and science news is not misleading, especially when it includes health advice for readers. News pieces have a different purpose to, and readership from, journal articles and are not expected to reproduce them or express claims in the same way. However, given that news is often explicitly or implicitly blamed for distorting and exaggerating scientific findings,^9^ it is pertinent to determine the sources of such misreporting. In fact there is little evidence on how often news stories go beyond what scientists state in peer reviewed journal articles, and, when they do, whether misrepresentation is already present in the un-peer reviewed sources supplied by scientists and press offices.

Previous research suggests that press releases can be a source of misinformation. Of 200 randomly selected medical press releases in 2005, 29% were rated as exaggerated and less than half provided appropriate caveats to their claims.^20^ In a study of 23 press releases and 71 associated news stories about cancer genetics, two thirds of claims in the press release were at least as deterministic as the claims in the news.^21^ However, since these studies did not compare press releases with statements made in the abstracts or discussions of the associated peer reviewed journal articles, they may not be examples of exaggeration beyond what journal articles routinely include themselves. Indeed, in a study on “spin” in the reporting of randomised controlled trials (70 press releases and associated journal abstracts, 41 news stories), in only four cases the news contained spin where the associated journal abstract did not.^22^

We aimed to clarify how often news contains claims or advice from health related research that go beyond those in the peer reviewed journal articles, and to identify the likely source of these exaggerations (press releases or news). Furthermore, we tested whether exaggerations in press releases were associated with a higher likelihood of news coverage, compared with press releases without exaggeration.

## Methods

From publicly accessible university repositories we identified all the press releases based on published studies with possible relevance for human health (biomedical and psychological sciences; fig 1) issued in 2011 by the Russell Group universities (the 20 leading UK research universities). We selected these universities as a clearly defined group with international prominence; we did not expect differences between this sample and other UK or international press releases (see for example^20^ ^21^). For each relevant press release (n=462) we sourced the associated peer reviewed journal article and print or online news stories (n=668) from national press using the Nexis database, BBC, Reuters, and Google (we did not include broadcast news; the number of news stories per press release ranged from 0-10). We coded each journal article, press release, and news set using a detailed protocol available online (http://dx.doi.org/10.6084/m9.figshare.903704; supplementary information SI sections 1-3 provide full details of our sample and methods). Each set took on average 3-4 hours to code. We double coded 27% of press releases and journal articles and 21% of news stories (concordance rate 91%, mean κ=0.88; given the large number our simulations show that 10% disagreement would not influence our conclusions, see supplementary section SI7).

**Figure fig1:**
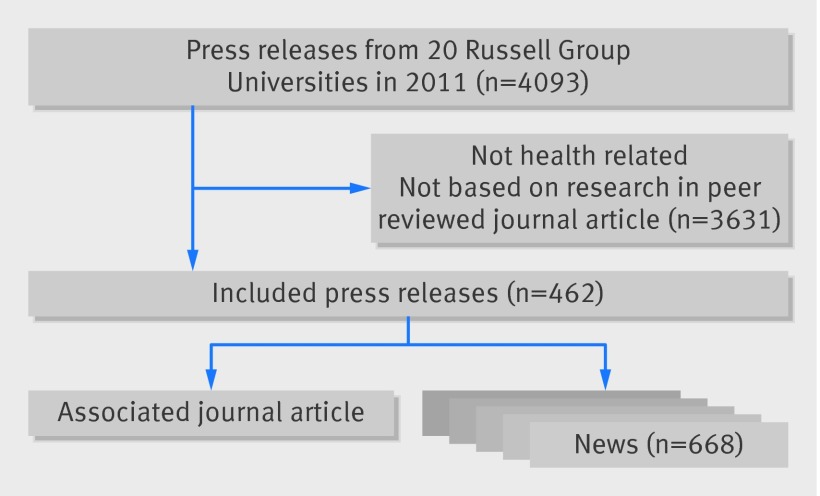
**Fig 1** Identification of press releases based on published studies with possible relevance for human health (biomedical and psychological sciences

Taking the peer reviewed paper as a baseline (which is not to assume that peer reviewed publications are true; many already contain exaggeration), we sought cases where news stories offered advice to readers, made causal claims, or inferred relevance to humans beyond (or different to) that stated in the associated peer reviewed paper. Given the likelihood that some statements in journal articles themselves would be considered exaggerated by other scientists in the specialty, our overall levels of measured exaggeration are likely to be underestimates. We then asked whether such discrepancies were already present in the corresponding press release. For example, if a study reported a correlation between stress and wine consumption and the news story claimed that wine causes stress, what did the press release say? Similarly, if a news story claimed a new treatment for humans but the study was on rodents, what did the press release say?

We focused our study on analysing advice to readers to change behaviour, causal statements drawn from correlational results (cross sectional and longitudinal observational data), and inference to humans from animal research.^23^ Explicit advice clearly has the potential to influence behaviour, as do causal claims about what factors influence health. It is notoriously difficult to ascertain cause from correlational results. For example, a correlation between consuming wine and a disease could occur because wine increases the risk of the disease, the disease increases the consumption of wine, or the consumption of wine correlates with another factor that is associated with the increased risk. For animal research, it is estimated that less than 10% of non-human investigations ever succeed in being translated to human clinical use.^24^ Over-selling the results of non-human studies as a promised cure potentially confuses readers and might contribute to disillusionment with science.^11^

### Advice

We coded each journal article, press release, and news story for the maximum level of advice it contained using four levels based on explicitness and directness: no advice, implicit advice (for example, “these findings suggest that mid-late childhood may be the best bet for childhood obesity prevention”, “simply exercising with a best friend or having a friend who is a good exercise role model increases the chance of a child keeping fit and active”), explicit advice, but not to the reader or general public (for example, “I think we now have enough evidence to say that pulse oximetry screening should be incorporated into everyday clinical practice”, “ambulatory monitoring is recommended for most patients before the start of hypertensive drugs”), and explicit advice to the reader or general public (for example, “children who are thirsty should be encouraged to drink water”, “for anyone considering taking aspirin I would recommend . . .”). Relevant samples for analysis of exaggeration of advice were those containing at least one implicit or explicit advice statement anywhere in the journal article or press release or news (n=213 press releases, n=116 press releases with news; n=360 news stories).

### Causal statements from correlational results

For journal articles, press releases, and related news stories associated with correlational results we coded for the strength of the main statements of the findings. For press releases and news we used the title and first two sentences as their main statements, since nearly all follow the “inverted pyramid” structure of stating their main claims first.^25^ For journal articles we used the abstract and discussion. We used a seven point scale to rate increasing levels of determinism, where the presence of stronger statements trumped weaker ones: no statement (in which case no further comparison was possible), explicit statement of no relation, correlational (for example, “drinking wine is associated with increased cancer rates”), ambiguous (for example, “drinking wine linked to cancer risk”), conditional causal (for example, “drinking wine might increase cancer risk”), can cause (for example, “drinking wine can increase cancer risk”), and unconditionally causal (for example, “drinking wine increases cancer risk”). For analysis of causal claims we focused on correlational research, which we defined as observational cross sectional and longitudinal designs. We did not analyse qualitative, interventional, or simulation designs. We coded the first claim statement for our primary analysis (relevant samples for analysis were 182 press release, 95 with news; 261 news stories). Where a second statement occurred about a different variable pair, we also coded these for replication (see supplementary section SI5 for analysis).

### Conclusions for humans from studies in non-humans

For each non-human study (animals, cells, or simulations), we coded whether the main statements of press release and news were phrased as explicitly non-human, implicitly human (for example, “a pregnant mother’s stress level affects the brain of her unborn baby”), or explicitly human (for example, “a pregnant woman’s stress . . .”). For journal articles we searched the discussion section and abstract for any statements about human relevance. Relevant samples for analysis were 105 press releases, 48 with news; 115 news stories.

### Caveats and justifications

We searched the whole press release and news stories for any caveats stated for the advice, causal claims, or inference to humans (for example, “This is a population study. It cannot say definitively that sugary drinks raise your blood pressure, but it’s one piece of the evidence in a jigsaw puzzle”, “The scientists who carried out the study emphasized that they could not say for certain . . .”). Similarly, we searched for justifications of the advice, claims, or inference (for example, “even after taking into account the effect of extra body weight on blood pressure, there was still a significant link with sweetened drinks”).

### Study facts and quotes

We also coded facts about the study and press release, including sample size, duration, completion rate, and the source of quotes. These are analysed in section SI11 of the supplementary file.

Further details of the coding methodology are given in section SI2 of the supplementary file. All coding sheets (n=462), full instructions for coding, and data analysis files and programs are available online (http://dx.doi.org/10.6084/m9.figshare.903704).

### Statistical analysis

We used generalised estimating equations to calculate percentages and 95% confidence intervals for news with exaggeration relative to what was present in the journal article, while adjusting for the clustering of several news articles to one press release (using an exchangeable working correlation). The generalised estimating equations framework was also employed to estimate the association (in odds ratios) between exaggeration in the press release and exaggeration in the news. Note that these analyses included only those journal articles and press releases for which there was at least one news story (and the news could be appropriately coded for the relevant analysis). We compare the characteristics of press releases with and without associated news, using bootstrapped 95% confidence intervals and standard inferential statistical tests.

## Results

### Exaggeration rates in press releases

For our analysis of advice we found that 40% of the press releases contained more direct or explicit advice than did the journal article (bootstrapped 95% confidence interval 33% to 46%). For our analysis of statements based on correlational results (cross sectional or longitudinal) we found that 33% of primary claims in press releases were more strongly deterministic than those present in the associated journal article (bootstrapped 95% confidence interval 26% to 40%). For studies on animals, cells, or simulations, 36% of press releases exhibited inflated inference to humans compared with the journal article (bootstrapped 95% confidence interval 28% to 46%). Given the likelihood that some statements in journal articles themselves would be considered exaggerated by other scientists in the specialty, our levels of measured exaggeration are likely to be underestimates.

### Association of news exaggeration with press release exaggeration

Figure 2 summarises the rates of exaggeration in news for press releases that did or did not already contain exaggeration. For advice, overall 36% of news (95% confidence interval 29% to 44%) contained more direct or explicit advice than did the journal article. The odds of exaggerated advice in news was 6.5 times higher (odds ratio 6.5, 95% confidence interval 3.5 to 12.4) when the press release contained exaggerated advice (58%, 95% confidence interval 48% to 68%; see table[Table tbl1] for the odds) than when it did not (17%, 10% to 24%; difference 41%, 95% confidence interval 28% to 53%).

**Figure fig2:**
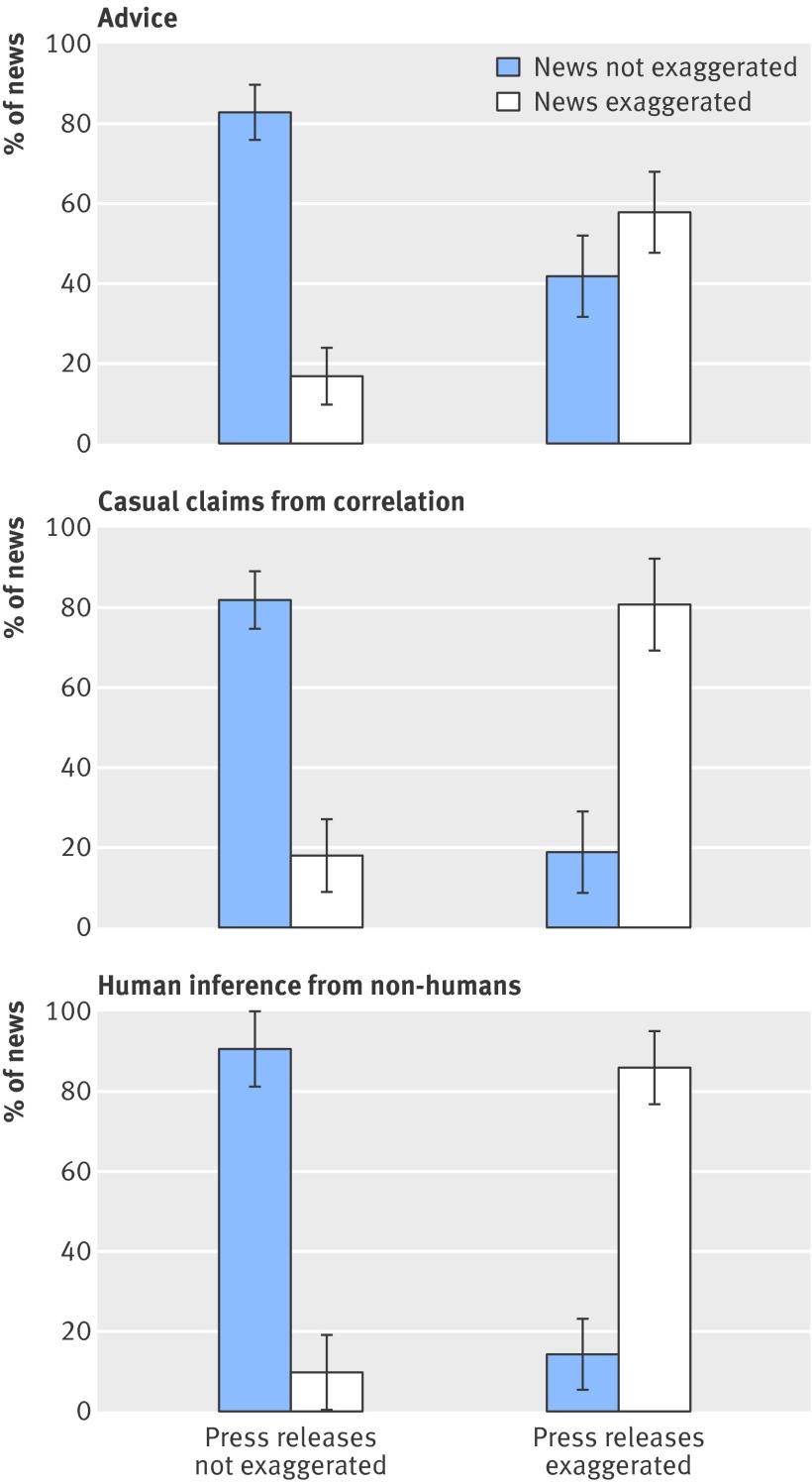
**Fig 2** Proportions of news with exaggerated advice, causal statements from correlational research, or inference to humans from non-human studies were higher when the associated press releases contained such exaggeration. Error bars are 95% confidence intervals. See table for odds ratios

**Table tbl1:** Summary of results for analyses of advice, primary claims from correlational data (causal claims), and human inference from non-human studies (human inference)

Variables	No	PR with news	No with news	Odds news uptake	Odds ratio (95% CI)	Odds news exaggerated	Odds ratio (95% CI)
Advice:							
PR not exaggerated	128	66	188	1.1	1.3 (0.8 to 2.4)	0.2	6.5 (3.5 to 12.4)
PRs exaggerated	85	50	172	1.4		1.4	
Total	213	116	360	—		—	—
Causal claims:							
PR not exaggerated	122	61	169	1	1.3 (0.6 to 3.0)	0.2	19.7 (7.6 to 51.4)
PRs exaggerated	60	34	92	1.3		4.3	
Total	182	95	261	—	—	—	—
Human inference							
PR not exaggerated	67	29	68	0.8	1.3 (0.7 to 2.5)	0.1	56.1 (14.9 to 211)
PRs exaggerated	38	19	47	1		5.9	
Total	105	48	115	—	—	—	—

PR=press release.

The key results are that odds ratios for the dependence of news uptake on PR exaggeration are indistinguishable from 1, whereas odds ratios for the dependence of news exaggeration on PR exaggeration are much larger and clearly distinguished from 1. See the results section and figures 2 and 3 for further information, including percentages and 95% confidence intervals.

For main news statements about correlational results, 39% (95% confidence interval 31% to 49%) were more strongly deterministic than those present in the associated journal article. The odds of exaggerated statements in news was 20 times higher (95% confidence interval 7.6 to 51) when press release statements were exaggerated (81%, 95% confidence interval 70% to 93%) than when they were not (18%, 9% to 27%; difference 63%, 95% confidence interval 49% to 78%).

For non-human studies, 47% of news contained inflated inference to humans. The odds of exaggeration in news was 56 times higher (95% confidence interval 15 to 211) when press release statements were exaggerated (86%, 95% confidence interval 77% to 95%) than when they were not (9.6%, 0% to 19%; difference 76%, 95% confidence interval 63% to 89%). See supplementary section SI5-7 for further details of these results.

### Effect of exaggeration in press releases on news uptake

A key motivation for inflating advice, causal inference, or inference to humans in press releases may be the assumption that it greatly increases news uptake. Contrary to our expectations, however, the proportion of press releases with at least one associated news story did not differ significantly between press releases with exaggeration and those without for any of our three analyses (figure 3 and table), although in this dataset we cannot assess what the news uptake would have been for identical press releases with and without exaggeration. While there was a small numerical increase in news uptake with exaggerated press releases, any real effect is unlikely to be greater than the upper confidence intervals. For advice, 66/128 (52%) press releases without exaggeration had news uptake compared with 50/85 (59%) press releases with exaggerated advice (bootstrapped 95% confidence intervals of the difference −6.4% to 21%). For causal claims from correlation, 61/122 (50%) press releases without exaggeration had news uptake compared with 34/60 (57%) press releases with exaggerated claims (95% confidence intervals of the difference −9% to 22%; see supplementary SI5 for secondary statements). For inference to humans, 29/67 (43%) press releases without exaggeration had news uptake compared with 19/38 (50%) press releases with exaggerated advice (95% confidence intervals of the difference −13% to 27%).

**Figure fig3:**
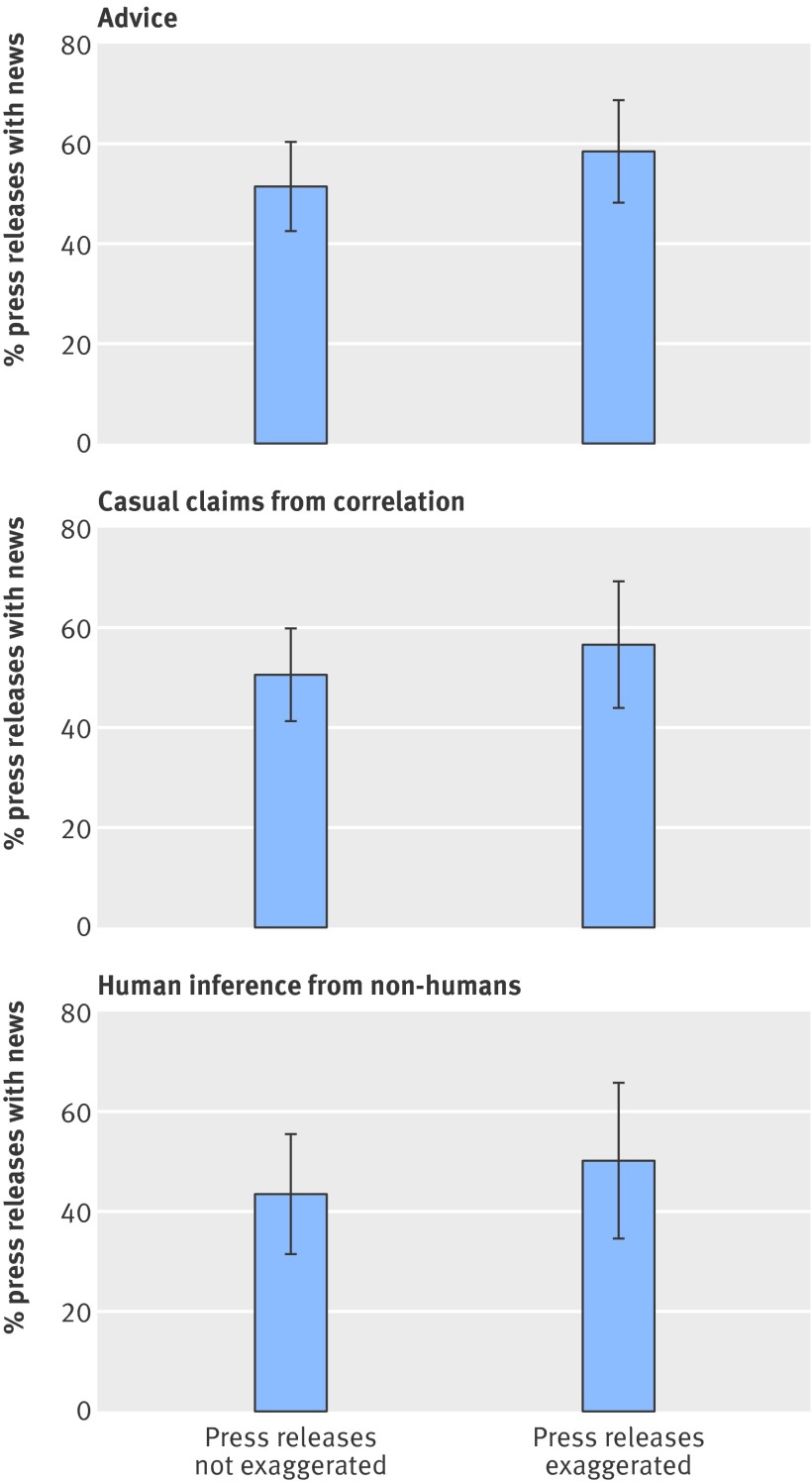
**Fig 3** The proportion of press releases with some news uptake (at least one news story) was not statistically distinguishable regardless of whether the press release did or did not contain exaggerated advice, causal statements, or inference to humans from animal research. Furthermore, the mean number of news stories per press release did not significantly differ with exaggeration (data not in figure, see text). Error bars are bootstrapped 95% confidence intervals. See table for odds ratios

Further, there was no statistical support for the idea that when press releases do successfully generate news, exaggeration would be linked with more associated news stories. As for percentage news uptake, any real effect is unlikely to be greater than the upper confidence intervals. Non-exaggerated advice was associated with 2.8 news stories per press release, whereas exaggerated advice was associated with 3.4 news stories per press release (95% confidence intervals of difference −0.3 to 1.5). Non-exaggerated main causal claims were associated with 2.8 news stories per press release, whereas exaggerated causal claims were associated with 2.7 news stories per press release (95% confidence intervals of difference −1.0 to 1.0). Non-exaggerated inference to humans was associated with 2.3 news stories per press release, whereas exaggerated inference was associated with 2.5 news stories per press release (95% confidence intervals of difference −0.8 to 1.1).

Between universities there was also no evidence that higher rates of inflated claims in press releases attracted more news uptake. The percentage of inflated advice, causal statements, or inference to humans in press releases varied from 11% to 50%, while the proportion of press releases with news varied from 8% to 87%, but these did not significantly correlate (r=0.13; see supplementary section SI9).

We also tested whether explicit caveats mentioned about advice, causal statements, or inference to humans from animal research in press releases were associated with reduced news uptake, as many scientists and press officers might fear. Overall, caveats were rare in press releases, and there was a clear association between their presence or absence in press releases and in news (see supplementary section SI8). But we found no evidence for an effect on uptake; if anything, caveats to causal statements might be associated with higher uptake (69% *v* 51%, bootstrapped 95% confidence intervals of difference −0.1% and 35%; note that numbers are small for caveats). We also coded the presence of justifications for advice, causal claims, and inference to humans that would help readers judge which statements are warranted; these were also rare, highly associated between press release and news, but with no evidence for an effect on news uptake (see supplementary section SI10).

## Discussion

Although it is common to blame media outlets and their journalists for news perceived as exaggerated, sensationalised, or alarmist, our principle findings were that most of the inflation detected in our study did not occur de novo in the media but was already present in the text of the press releases produced by academics and their establishments. Among biomedical and health related press releases issued by Russell Group universities in 2011, 33% to 40% contained exaggerated statements compared with the corresponding peer reviewed journal articles. Moreover, when press releases contained exaggeration it was likely that the news would too (58% for advice, 81% for causal claims, and 86% for inference to humans, fig 2), but when press releases did not contain exaggeration, rates of exaggeration in news were only 17%, 18%, and 10%, respectively. Therefore the odds of exaggerated news were substantially higher when the press releases issued by the academic institutions were exaggerated (odds ratios 6.5, 20, and 56, respectively).

### Caveats for our observational approach

Our study was correlational, so does not demonstrate a causal relation between inflated statements in press releases and inflated news. For example, if journalists did not read the press releases, associated exaggeration could nevertheless emerge between the press release and news because of features in the journal articles that might naturally lead to such exaggerations. However, many sources of converging evidence point to press releases as the main source of science news,^17^ ^18^ ^21^ ^26^ ^27^ including the quotes and study facts analysed from our data (see supplementary section SI11). Although some of the studies will have had press releases released from both university and journal, this could only increase the proportion of occasions when exaggeration is already contained in at least one important press release source. It is not yet known whether exaggeration rates in press releases issued by journals differ noticeably from those issued by universities; our ongoing research is exploring this further.

Changes in presentation style between peer reviewed papers and press releases are expected in order to spark the interest of journalists. But seeking simplification and stimulating interest does not justify exaggeration. Moreover, contrary to common assumption, we did not find evidence that exaggerated statements in press releases are more likely to attract news uptake or substantially increase the number of news articles when they do occur. We also found no indication that caveats in press releases reduce uptake, although presumably the fear that they do is the reason caveats are so rare. These aspects of our results should be clarified by further research. It may not be simply the case that similar press uptake would be achieved with non-exaggerated headlines and inclusion of caveats. For example, press releases with exaggeration may not be based on journal articles with news value equal to those without exaggeration. Similarly, caveats may have been included in our sample of press releases only where likely press interest was already judged to be sufficiently strong.

### Using journal articles as the baseline

Since we are not experts in every discipline (and experts also disagree), we did not attempt to code whether changes to advice, causal claims, and inference to humans from animal research were scientifically justified. It is possible that some journal articles are worded over-cautiously, and in these cases stronger or more direct statements in press releases might be justifiable (although our results showed that they are rarely explicitly justified in press releases, see supplementary section SI10). However, we assume that pressure to publish means that most journal articles already contain the highest level (at least) of justifiable inference and advice; if further inflation occurs in a press release, it is thus likely to go beyond what a consensus of scientific opinion would find acceptable. Consistent with this interpretation, a preliminary survey (see supplementary section SI12 and figure S2) revealed that a surprising number of scientists were willing to say that their press releases were exaggerated (relative to their own judgment of what was scientifically justified). Furthermore, given the imperfections of peer review, many journal articles may contain statements that are already exaggerated relative to a consensus of scientific opinion, or at least spun to emphasise positive findings,^22^ and thus our measured level of within university exaggeration is likely to underestimate the extent of the problem.

### Implications for practice

It is important that these results are not perceived as simply shifting the blame from one group of non-scientists (journalists) to another (press officers). Most press releases issued by universities are drafted in dialogue between scientists and press officers and are not released without the approval of scientists^20^ (and confirmed in our survey, see supplementary section SI12), and thus most of the responsibility for exaggeration must lie with the scientific authors. At the other end of the chain, journalists have a continuing responsibility to cross check their sources even if their working conditions make that increasingly difficult. The blame—if it can be meaningfully apportioned—lies mainly with the increasing culture of university competition and self promotion, interacting with the increasing pressures on journalists to do more with less time. It is interesting in this context that news outlets were broadly similar in the degree of exaggeration between press release and news (see supplementary section SI13).

Our findings may seem like bad news but we prefer to view them positively: if the majority of exaggeration occurs within academic establishments, then the academic community has the opportunity to make an important difference to the quality of biomedical and health related news. Arguably it would be far more difficult to change the working practices and cultures of journalists at independent news organisations. Furthermore, we are not arguing that accurate (or appropriately cautious) claims are sufficient for the public readership to make well informed choices in health related issues (that is, the discredited information deficit model).^19^ The potential influence of the media on the opinion and behaviour of different publics is complex and other factors are involved.^1^ ^5^ ^6^ What we do argue is that appropriate claims are a necessary starting point, that misleading claims can do harm, and that since many such claims originate within universities, the scientific community has the ability to improve this situation.

**What is already known on this topic**

Health related news has widespread potential to influence health related behavior but often misreports the science
It is not known whether exaggerations originate in the news stories themselves or in press releases issued by academic institutions producing the research

**What this study adds**

Most exaggeration in health related science news was already present in academic press releases
Exaggeration was not significantly associated with increased news coverage, relative to press releases without overstatement
Press releases could be a primary target to improve the accuracy of science news, with potential benefit for public health

## Notes

We thank our professional advisory group for input into this research, including Rob Dawson (BBSRC), Fiona Fox (Science Media Centre), Ruth Francis (Biomed Central), James Gallagher (BBC), Helen Jamison (Science Media Centre), Alok Jha (*The Guardian*), Emma Little (*The Sun*), Carmel Turner (Medical Research Council), Bob Ward (Grantham Institute), and Ed Yong (*National Geographic*); Ananyo Bhattacharya and Simon Dymond for helpful discussions; and Eleanor-Rose Corney and Caitlin Argument for research assistance.

Data sharing: All coding sheets (n=462), full instructions for coding, summary data files, and analysis programs are available online (http://dx.doi.org/10.6084/m9.figshare.903704).

Transparency: The lead authors (PS and CDC) affirm that the manuscript is an honest, accurate, and transparent account of the study being reported; that no important aspects of the study have been omitted; and that any discrepancies from the study as planned have been explained.

## Notes

Cite this as: *BMJ* 2014;349:g7015
