# Supplementary material for: The association between exaggeration in health related science news and academic press releases: retrospective observational study
Source: BMJ. 2014 Dec 10;349:g7015. doi: 10.1136/bmj.g7015 (PMC4262123; doi:10.1136/bmj.g7015)
Supplement: Supplementary file 1 — Supplementary information [file sump020572.ww1_default.pdf]

The association between exaggeration in health-related science news and academic press releases: retrospective observational study

Supplementary Information

List of acronyms

DV, Dependent variable; IV, Independent variable; JA, Journal article; PR, Press release.

SI1. Materials

Press releases (PR). PRs from Russell Group members (in 2011) were obtained from the online press site of each university (University of Birmingham, University of Bristol, University of Cambridge, Cardiff University, University of Edinburgh, University of Glasgow, Imperial College London, King's College London, University of Leeds, University of Liverpool, London School of Economics, University of Manchester, Newcastle University, University of Nottingham, University of Oxford, Queen's University Belfast, University of Sheffield, University of Southampton, University College London, University of Warwick). All press releases published in 2011 were inspected, and those not relating to human health, psychology, or neuroscience were excluded. Also excluded were those about future research not yet performed.

Journal articles (JA). The JAs corresponding to each press release were obtained in several ways. Some PR contained a full link; others reported the lead author name, and either the article title, journal name or both. In all such cases, an online search identified the article. In cases where only the lead author name was mentioned, the author's full publication list was found on the university web page, and each publication in 2010, 2011 and 2012 was inspected until the JA corresponding to the PR could be identified. If the JA could not be identified (usually after 2-4 hours of search), that PR was excluded from the analysis. For 6 PRs there were two associated JAs, and in 1 case 3 JAs.

News stories. News sources included were: *Associated Press, BBC News, Daily Mail, Daily Star, Daily Star Sunday, Economist, Express, Financial Times, Guardian, Guardian.co.uk, Independent, Mail on Sunday, Mail Online, Metro, Mirror, New Scientist, Observer, People, Press Association, Racing Post, Reuters, Scotsman, Sun, Sunday Express, Sunday Mail, Sunday Mirror, Sunday Post, Sunday Sun, Sunday Telegraph, Sunday Times, Telegraph, Times.*

First, the key words of the press release were searched on the Nexis® database (http://www.lexisnexis.com/uk/nexis/). The search was limited to UK national newspapers and articles published within 30 days of the PR. Each search was performed several times with different key words, from specific to more general. For example, for the PR titled "Scientists demonstrate potential new treatment for most common form of infant leukaemia", the search would be performed with key words "BET proteins AND MLL", "proteins AND leukaemia", "new treatment AND leukaemia", and "treatment AND cancer". In the same way, BBC news webpages (http://www.bbc.co.uk/news/) and Reuters webpages (http://uk.reuters.com/) were searched. Finally, a google search was performed using the key words and the name of each individual newspaper. In cases where several news articles were found from the same newspaper, the longer article was selected for coding.

SI2. Coding

The coding template and full instructions for coders are available online (http://dx.doi.org/10.6084/m9.figshare.903704). These were designed by the authors to address the three main questions of interest (advice to readers, causal conclusions from correlation, and generalizations from non-human samples to humans). It was developed using a pilot batch of materials by iterative expansion and modification in order to capture aspects relevant to our main questions that were not adequately coded initially. Pilot testing was used to clarify potential ambiguity, and to solve coding difficulties and instances of low inter-rater reliability.

Each set of JA, PR and news took 3-4 hours to code on average, but varied depending on clarity of the materials, number and length of news articles and complexity of the study.

Advice. The PR, news stories and JA (abstract and discussion) were read for statements of implicit or explicit advice. Coding levels were:

0, *No advice* (including advice to researchers, for example to perform further study).
1, *Implicit advice* (e.g. "Eating chocolate might be beneficial for... ").
2, Explicit but not to reader or general public (e.g. "Doctors should advise patients to...")
3, Explicit to reader / general public (e.g. "Expectant mothers should... ").

A range of examples of inflation is given below (note that not all would be considered inappropriate if they are just changes to the intended audience; our purpose is not to evaluate each inflation, but to find the source given that inflation is generally blamed on journalists). PR: *Mothers who want to breastfeed should be given all the support they need* (code 2); News: *Mums should breastfeed for at least four months to avoid having naughty kids, experts now advise* (code 3). PR: *If these weather patterns continue, both forage and dairy management will have to adapt to maintain current milk quality* (code 1); News: *spend 9p extra a pint and save Daisy the Dairy Cow, in her straw hat* (code 3). JA: *the data we present add to growing justification to monitor the health of preterm men and women beyond infancy and childhood* (code 1); PR: *we need to monitor the health of premature babies beyond infancy and childhood* (code 2). JA: *These specific defects should be included in public health educational information to encourage more women to quit smoking* (code 2); PR: *women should quit smoking before becoming pregnant, or very early on, to reduce the chance of having a baby with a serious and lifelong physical defect* (code 3). PR: *It is possible that good nutrition during the first three years of life may encourage optimal brain growth* (code 1); News: *People should seek advice from a registered dietician, but simply it's a message of moderating fat intake, five fruit and veg a day and whole grain starchy foods* (code 3). PR: *Our findings support the concept of more widespread HIV testing* (code 1); News: *if you've been at risk for HIV, get tested now* (code 3).

Causal statements from correlational research. For each PR and news story, the IV (or pseudo IV in correlational designs), DV and stated relationship between them (if any) were extracted from the main claims, which were operationalized as the title plus first two sentences in PRs and news. For the JA, main claims were defined within the abstract and discussion sections. If there were claims about more than one set of IV and DV in the PR or news, a second set was also coded and the same sets were identified in the JA, allowing us to test whether the findings for the main statements are replicated in the second statements (SI5).

In order to consistently code the 6 levels of relationship statement we drew up a table of examples from the first stage of coding. These were:

0. No relationship stated (but could have been): The study must have contained at least two variables (IV and DV, or pseudo IV and DV) between which a relationship could have been stated. If there were not two suitable variables, the code 'not applicable' was used.
1. Statement of NO relationship/cause: e.g. 'no difference'; 'persists without'; 'does not result in'; 'no significant extra risk'; 'added no benefit'.
2. Statement of correlation: Meaning remains if the variables in the statement are reversed; e.g. 'associated with'; 'related to'; 'varies with'; 'increases as'; 'decreases as'; 'have higher/lower rates of'; 'linear relationship';
3. Ambiguous statement of correlation/cause: Strictly does not state cause, but contains stronger implication of direct relationship than level 2; e.g. 'linked to'; 'predicts'; 'connected to'; 'more successful'; 'had difficulty';
4. Conditional cause: Causal statement (examples in level 6) preceded by conditional, such as 'might', 'could', 'may', 'appears'; or a statement that implies 'might cause', e.g. 'likely to be a critical event', 'demonstrates potential'.
5. Can cause: Causal statement (examples in level 6) preceded by 'can'. 'Can cause' is more deterministic than 'may cause' because it denotes ability, and implies 'does sometimes', while 'may' denotes a potential not yet realised. Note that although 'could' and 'might' are grammatically related to 'can' and 'may', respectively, 'could' is closer in meaning to 'might' in common usage than 'can' is to 'may'. Thus we grouped 'could' with 'might' in category 4.

6. Causal statement: Direct causal statement; e.g. 'enhances', 'increases' [as transitive verb - X increases Y], 'decreases', 'cuts', 'ameliorates'; 'influences'; 'prevents'; 'raises/reduces'; 'enables'; 'determines'; 'is vital for'; 'boosts/improves'; 'drives'; 'leads to'; 'transforms'; encourages' etc.

Where statements of different levels were made within the analysed segments of text, stronger statements trumped weaker ones. Separately, we also coded whether or not the statement of relationship was explicitly probabilistic – for example, 'correlated with the risk of...' (correlational probabilistic); 'raises the chance of...' (causal probabilistic); Further probabilistic words/phrases included: 'likelihood'; 'makes more likely'; 'tendency'; 'rate'.

For analysis of causal claims we focused on correlational research; we coded type of study design using 6 categories: Qualitative; Correlational cross-sectional; Correlational longitudinal; Intervention (not full RCT); Full randomised controlled trial (RCT); Modelling / Simulation. We did not detect any differences in the distribution of causal statement levels between cross sectional and longitudinal correlational designs; therefore we grouped these together into a single correlational category for further analysis. We did not analyse qualitative, interventional or simulation studies further. We checked whether the IVs and DVs themselves got distorted, changed or generalised in the progression of claims. We find that this happened in PR for IVs in only 11/573 samples, and for DVs in only 6/554; similarly in news only 21/726 for IVs and 11/740 for DVs.

A range of examples of inflation is: JA: *This observational study found significant associations between use of antidepressant drugs and several severe adverse outcomes in people aged 65 and older with depression* (code 2); PR: *New antidepressants increase risks for elderly* (code 6). JA: *Reported flooding experiences had a significant relationship with perceptions relating to climate change* (code 2); PR: *Direct experience of extreme weather events increases concern about climate change* (code 6). JA: *A brief TCBT or exercise program was associated with substantial, significant, clinically meaningful improvements in self-rated global health* (code 2); PR: *Talking therapy over the phone improves symptoms of chronic widespread pain* (code 6). JA: *deregulation of a single kinase in two distinct cellular compartments... is intricately linked to implantation failure and miscarriage* (code 3); News: *The protein SGK1 in the lining of the womb makes it harder to get pregnant* (code 6). JA: *bisphosphonate use is associated with a significantly lower rate of revision surgery of up to about 50% ... in patients without a previous fracture* (code 2); News: *Bisphosphonates 'extend hip replacement life'* (code 6). JA: *human orbital volume significantly increases with absolute latitude* (code 2); News: … *gives you a bigger brain* (code 6). JA: ... *association between RXRA chr9:136355885+ methylation and mother's carbohydrate intake* (code 2); PR: *During pregnancy, a mother's diet can alter the function of her child's DNA* (code 5).

Human conclusions from non-human studies. We coded the explicit or implicit study sample, population type or experimental participants of the main claims in JA, PR and news. We used the same code to separately identify the actual sample, population type or experimental participants of the study. If there was more than one type (e.g. rodent and human) in a JA, it was excluded from the analysis of human inference from non-human studies. The coding levels were:

1. Explicitly human: explicit mention of e.g. *humans*, *people, participants, women, men, girls, boys*; pronouns e.g. *we*, *our*, *your*; mention of specific study designs e.g. *genome-wide association study*, *prospective cohort study;* mention of activities that relate to humans only, e.g. *mobile phone use*, *reading books*, *A levels.*
2. Implicitly human: no explicit mention of sample, yet the sentences are judged to refer to humans, e.g. *eating chocolate causes cancer*, or *new drug found for Alzheimer's*
3. Non-human primates: explicit mention of *chimpanzees*, *monkeys*, etc.
4. Rodents: explicit mention of *mice*, *rats*, *rodents*
5. Other animals/organisms: explicit mention of *fruit flies*, *bacteria*, *worms*, *birds*, *fish*, etc.
6. Cells *in vitro*: explicit mention of cell cultures, e.g. *stem cells*, *T cells*, etc.
7. Simulations: explicit mention of *mathematical modelling* or *computer simulations* etc.

A range of examples of inflation is: JA: *We have developed a broadly effective strategy to polymerize monomeric Fc-fusion proteins...* (code 6); PR: *Better and more affordable treatments for sufferers of autoimmune diseases* (code 1). JA: *An animal model of this important component of memory consolidation...* (code 4); PR: *Scientists have shed light on why it is easier to learn about things related to what we know* (code 2). JA: *In mouse tooth development, Barx1*

expression is restricted to presumptive molar mesenchyme and throughout tooth development to molar mesenchyme cells (code 4); PR: *Researchers have uncovered a novel mechanism they have termed 'developmental stalling', that might explain how errors in the development of human embryos are naturally corrected to prevent birth defects* (code 2).

Caveats and justifications. For each section, we searched the whole PR and news stories for any caveats stated for the advice or claims (e.g. "This is a population study. It can't say definitively that sugary drinks raise your blood pressure, but it's one piece of the evidence in a jigsaw puzzle"; "The scientists who carried out the study emphasised that they could not say for certain..."). Similarly, we searched for justifications of the advice or claims (e.g. "even after taking into account the effect of extra bodyweight on blood pressure, there was still a significant link with sweetened drinks").

Study facts and quotes. We also coded various facts about the study and PR, including sample size, duration, completion rate and the source of quotes. These are analysed in section SI11 (Indicators of news sources).

SI3. Inter-rater reliability.

We double-coded 27% of PR and associated JA, and 21% of news stories. This difference is due to the fact that the PRs randomly selected for double coding had lower than average number of news stories. Inter-rater concordance was 90.5% (κ = .87) for cells relevant for the advice analysis; 86.3% (κ = .84) for cells in the analysis of causal claims and 94.4% (κ = .93) for cells analysed for human inference from non-human research. We analyzed the distribution of coding disagreements where they arose in the double-coded samples (i.e. whether each disagreement was between a code 1 and 2, or between a code 2 and 3 etc). Then within each round of the simulations in section SI7, 10% of the samples were by chance changed to another code in line with the observed distribution of coding disagreement in the double coded samples. This had a negligible effect on our results.

SI4. Association between advice, causal statements and human inference.

Of the studies contributing to the analysis of advice, 110 were included in the analysis of causal claims from correlation, while 19 were non-human studies included in the human inference analysis. There were only 14 studies that were both non-human and correlational. Thus while the analyses of advice and causation share many PRs, JAs and News, the analysis of nonhuman studies is on a largely independent sample of PRs, JAs and news.

Within the 110 correlational studies included in the advice analysis (because some level of advice was offered somewhere in JA, PR or news), the level of advice was not correlated with the level of causal claim within JA, PR or news (r=0.02, 0.05 and -.003, respectively). Within the 19 non-human studies included in the advice analysis (because some level of advice was offered somewhere in JA, PR or news), the level of advice was not significantly associated with the level of human inference (r=0.07, p=0.78; r=0.29, p=0.23; r= -0.29, p=0.12; although note that N in this analysis is small).

SI5. Secondary Statements (i.e. about a second set of variables in correlational studies)

For the secondary statements 25% (95% CI: 18-34%) were more strongly deterministic than those present in the associated JA. The odds of exaggerated statements in news were 36 times higher (OR=36, 95% CI: 7.8-148) when PR statements were exaggerated (83%, 95% CI 65-100%) than when the PR was not exaggerated (12%, 95% CI: 3.2-22%; difference=70%, 95% CI: 51-90%). Thus while secondary statements tended to be exaggerated less often (presumably because they are not the leading eye-catching statement), the association between exaggeration in PR and news is still very strong, replicating the results for main statements.

For rates of news uptake, 44/76 (58%) PRs without exaggeration had news uptake vs 13/26 (50%) PRs with exaggerated claims (bootstrapped 95% confidence intervals of the difference are -30% to +15%). Non-exaggerated secondary causal claims were associated with 3.0 news stories per PR, while exaggerated causal claims were associated with 2.2 news stories per PR (confidence intervals of the difference are -1.8 to +0.3).

SI6. Breakdown of PR exaggeration for exaggerated news

In the main analysis we categorized news and PR as exaggerated or not relative to the JA. This simple categorization did not distinguish between PRs that are exaggerated to the same extent as news and PRs that are exaggerated a bit, while the news is exaggerated further. In fact the latter case was relatively rare, and the most common scenario was for an identical level of exaggeration in PR and news. In the cases where news went beyond what was written in the JA, Figure S1 shows the proportions of cases when the associated PR contained no exaggeration relative to the JA (left solid bars in each plot, labeled PR ≤ JA) or when the PR did contain exaggeration relative to the JA (the three rightward solid bars in each plot, labeled PR>JA). Within the cases where the PR went beyond the JA (PR>JA), we plot the proportions when the news was further exaggerated from the PR (N>PR), when the news had equivalent statements to the PR (N=PR) and when the news was deflated again from the PR (N<PR, but remember news is still inflated relative to JA in order to qualify for this analysis). The key results are that we consistently found the largest category to be 'N=PR'; in other words, when the news was inflated relative to the JA, the most likely scenario for the PR was that it said the same as the news.

By adding this category (N=PR) to cases where PR was even more inflated than news (N<PR), we find that the PR was at least as inflated as the news on 70% (advice), 48% (causal claims from correlation) and 75% (human inference from non-humans) of cases. The by adding in the cases where there was some inflation from JA to PR, and then some more from PR to news (second bar, N>PR and PR>JA), we find the overall inflation rates occurring between JA and PR (78%, 75%, 90%). On the other hand, the inflation occurring between PR and news (30%, 52%, 25%) can be obtained from adding the two leftward columns: PR ≤ JA (remember all cases in this analysis have inflation from JA to news) and N>PR. Thus the rate of inflation between JA and PR consistently outweighs the rate of inflation between PR and news.

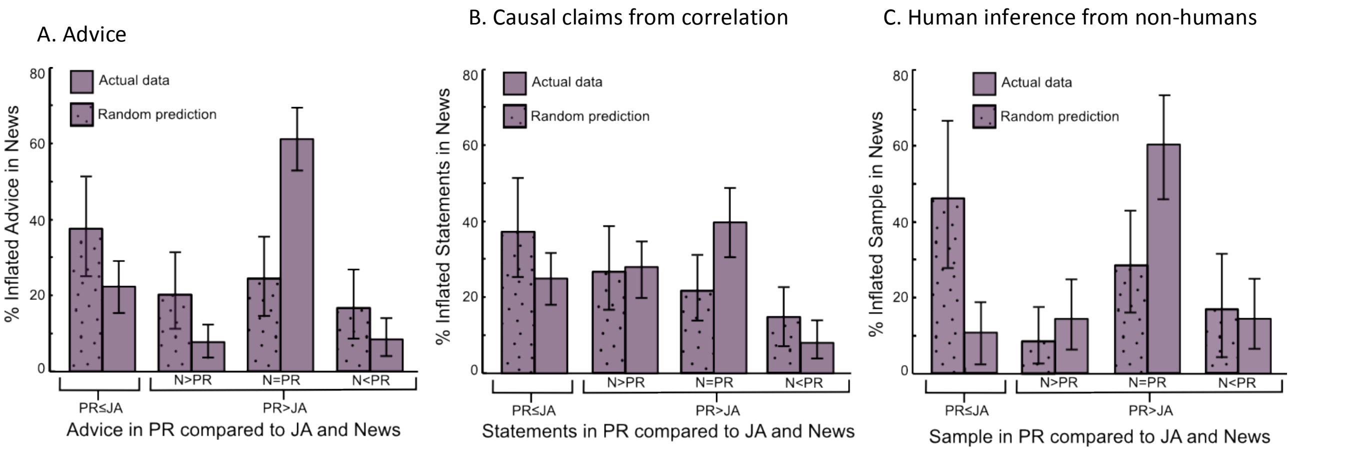

Figure S1. PR content where news contained exaggerated statements relative to the JA (N=131, 173, 49, respectively). In each plot, left bars (PR≤JA) indicate the cases where the PR contained nothing stronger than the JA. The other bars (PR>JA) indicate the cases where the PR contained inflated advice or statements relative to JA, in which case there could be further inflation in the news (N>PR), the same strength in news and PR (N=PR), or occasionally, deflation from PR to news (N<PR). Error bars show bootstrap-estimated 95% confidence intervals (the bootstrapping preserved the clustering structure of news to PR). The consistently most frequent situation in each analysis (**A**-**C**) was that the PR and news were equivalent, occurring much more often than chance prediction (dotted bars and associated error bars; advice, p<0.001; causal claims, p<0.001; human inference, p<0.001), estimated through simulating how often the observed distributions of coded levels in PR and news if written independently would produce each category plotted (see SI7). Adding the two rightmost bars together gives the proportion of cases where the PR was at least as inflated as the news (70%, 48% and 75% for A, B and C), while adding all three PR>JA bars together gives the proportion of occasions that there was some degree of inflation in the step from JA to PR (78%, 75%, 90%). For comparison, adding the two left bars together gives the total proportion of cases where there was some inflation from PR to news (30%, 52%, 25%).

SI7. permutation simulation of chance associations

Table S1 presents the distributions of coded advice levels for each category of outlet. We simulated the expected number of times that chance selection from these distributions would lead to the four categories displayed in Figure S1: no inflation in PR but inflation in news; PR inflated from journal article and news inflated further; the same level of inflation in both PR and news; and news inflated relative to journal article but deflated relative to PR. For each of 10000 iterations, the JAs, PRs and news were randomly reordered with respect to each other, but preserving the distributions shown in Table S1 and the clustering structure of news to PRs (i.e. that more than one news article can come from the same PR), and the analysis was rerun to categorize the inflation level in PR when there was inflation in news, just as for the analysis of the actual data in Figure S1. We also incorporated an estimate for the effect of coding dis-concordance (see SI3). To do this, we analyzed the distribution of coding disagreements where they arose in the double-coded samples (i.e. whether each disagreement was between a code 1 and 2, or between a code 2 and 3 etc). Then within each round of the simulation 10% of the samples were by chance changed to another code in line with the observed distribution of coding disagreement in the double coded samples. Adding this effect of coding dis-concordance had a negligible effect on results.

Similarly for correlational/causal claims and for human/non-human claims, we performed equivalent simulations based on the distributions of each statement level found in each outlet (Tables S2 and S3), the clustering structure of news to PR and the observed coding disagreement distributions in the double coded samples.

Note that the comparison of the actual number of cases where PR=news to these permutation analyses is conservative, since the simulations are likely to overestimate the chance expectation of PR=news. This is because they are based on distributions for each outlet that are not, in fact, independent. If they were independent, the similarity between the distributions would likely be reduced and this in turn would reduce the estimate of the associations that would occur by chance. In the extreme of non-independence, where most news stories were to copy a restricted range of phrases in PR, the estimated chance overlap would be very high due to the paucity of potential alternative options for the random sampling. In other words, since the occurrence of coding levels is not evenly distributed, as the real overlap between PR and news becomes larger, this simulation approach stacks the cards against finding differences between the data and the simulation. Thus we can be confident that where a statistically significant difference between the data and the chance simulations can be detected despite this bias, that difference is meaningful.

Table S1. The distribution (absolute N) of coded advice levels for each category of outlet, where there was some advice in at least one of JA, PR and news.

	No Advice	Implicit	Explicit not to the reader	Explicit to the reader
Journal article	75	79	52	7
PR	42	87	48	36
News	111	93	62	71

Table S2. The distribution (absolute N) of coded levels of causal/correlational statements about correlational studies for each category of outlet.

	No statement	No relationship	Correlational	Ambiguous	Conditional	Can	Causal
JA	14	9	96	18	7	6	64
PR	29	12	44	29	20	11	69
News	24	17	58	28	35	16	135

Table S3. The distribution (absolute N) of coded levels of human inference in non-human studies for each category of outlet.

	Non-human	Implicit Human	Explicit human
Journal article	96	3	6
PR	59	13	33
News	58	27	43

SI8. Predictors of news uptake

As shown in Figure 3 (main manuscript), inflation of advice, causal claims or human inference was not reliably associated with a higher proportion of PRs attracting news or a higher mean number of news per PR. That analysis compared inflated to non-inflated statements irrespective of the actual coded level of those statements (many strong statements are not inflated because they are also contained in the JA). While inflation was our main interest, we can also analyze whether simply the coded level of PR statements (irrespective of whether they were inflated relative to JA) was associated with news uptake. The proportion of PRs with news appeared to be about 15% greater where explicit advice was present, though this was not statistically significant even without correction for multiple comparisons ($\chi^2(3)=6.1$, p=0.11). There was even less indication that the proportion of PRs with news was predicted by the strength of main causal claims ($\chi^2(6)=2.6$, p=0.86), or human inference ($\chi^2(2)=3.2$, p=0.2). Relatedly, for the mean number of news stories per PR, explicit advice was associated with an increase only if uncorrected for multiple comparisons (F(3,458)=2.95, p=.03; no advice 1.3, implicit advice 1.4, explicit advice not to the reader 2.0, explicit advice to the reader 2.1). Likewise, for stronger human inference there was a possible increase that would not survive the appropriate correction for three comparisons (F(2,405)=3.86, p=0.02 uncorrected). There was no indication of an increase with stronger causal claims (F(6,207)=0.7, p=.7 uncorrected). Thus overall there is some suggestion, as would be expected, that stronger advice with relevance to humans attracted more news coverage, but these effects were, perhaps surprisingly, not strong enough to be clearly significant.

SI9. Comparison between universities

Table S4 shows the number of PRs from each university included in our analyses of advice, causal claims and human inference (without double counting those included for more than one analysis), as well as the percentage of claims that were un-inflated and the percentage that had news uptake. Due to the low numbers once broken down by university, advice, main causal claims, secondary causal claims and human inference are added together to form one category of 'statements', which is why N statements differs from N PR. Note that the percent of statements *without* inflation is given (rather than *with* inflation) for straightforward multiplication with % news uptake in the combined score that estimates the % of non-inflated PR attracting news. The table is ordered by % uninflated PR claims in order to illustrate the lack of any correlation with the % news uptake (r=-.13). Note that in 10 cases identical press releases were released by two universities on the same research; these are included here for each university, but they were not double counted in Figure 1, or in the analyses for Figs 2 and 3.

The ranks of inflation and uptake are also shown, as well as a rank for the estimated % non-inflated PR attracting news. However, it is important to note the relatively large confidence intervals on these ranks. Ranks alone often leave it difficult for the reader to discern whether a rank order is clear-cut or largely due to small differences and random variation. We estimated the confidence intervals using the following procedure: For Birmingham, we drew [Birmingham N] times with replacement from the pool of [Birmingham N] relevant Birmingham PRs and calculated the percent inflation and uptake; then for Bristol we drew [Bristol N] times with replacement from the pool of [Bristol N] relevant Bristol PRs and calculated percent inflation and uptake; and so on for each university. Rank orders for inflation, uptake and combined scores were then found to produce a table for that round of resampling. This procedure was repeated 100000 times to create 100000 tables, from which the 95% confidence intervals

for inflation rank, uptake rank and overall rank were estimated. The CIs are generally wide, partly because of the low N for some universities.

In the case of animal research, one possible reason for PRs to generalise to humans might be to avoid advertising animal research facilities. There is currently an impetus for scientists and institutions to be more open about animal research. Recently, a 'concordat on openness on animal research' was launched, supported by 16/20 of our sample universities: University of Birmingham, University of Bristol, University of Cambridge, Cardiff University, University of Edinburgh, University of Glasgow, Imperial College London, King's College London, University of Leeds, University of Manchester, Newcastle University, University of Nottingham, University of Oxford, Queen's University Belfast, University of Sheffield, and University College London.

Table S4. Rates of inflation and news uptake for health-related PRs from each Russell Group university in 2011 (see text above for explanation).

	N PR	N statements	N inflated statements	% without inflation	Inflation rank (95% CI)	% PR with news	Uptake rank (95% CI)	Combined score	Combined rank (95% CI)
**University of Oxford**	28	28	3	89%	1 (1-4)	54%	11 (5-16)	48%	3 (1-10)
**University of Warwick**	16	24	4	83%	2 (1-7)	56%	6 (4-16)	47%	4.5 (1-13)
**University of Manchester**	28	30	7	77%	3 (2-11)	43%	16 (8-18)	33%	11 (5-17)
**University of Liverpool**	12	15	4	73%	4 (1-17)	8%	20 (19-20)	6%	20 (18-20)
**London School of Economics**	5	10	3	70%	5 (1-19)	80%	3 (1-13)	56%	2 (1-13)
**University of Leeds**	17	26	8	69%	6 (3-17)	47%	14 (5-18)	33%	12 (4-18)
**University of Sheffield**	11	12	4	67%	7 (2-19)	55%	9 (3-18)	36%	8 (2-17)
**Imperial College London**	41	55	19	65%	8 (5-17)	54%	10 (5-15)	35%	10 (5-15)
**University of Southampton**	13	20	7	65%	9 (3-19)	62%	5 (3-16)	40%	7 (2-16)
**University College London**	15	17	6	65%	10 (3-19)	87%	1 (1-4.5)	56%	1 (1-8)
**University of Nottingham**	29	42	15	64%	11 (5-18)	38%	17 (10-19)	24%	16 (9-19)
**University of Cambridge**	45	53	19	64%	12 (5-17)	56%	7.5 (5-14)	36%	9 (5-14)
**University of Bristol**	52	73	27	63%	13 (6-17)	31%	18 (15-19)	19%	18 (14-19)
**University of Edinburgh**	36	40	15	63%	14 (5-18)	75%	4 (2-6.5)	47%	4.5 (2-10)
**University of Glasgow**	29	44	17	61%	15 (6-18)	52%	12 (5-16.5)	32%	13 (6-17)
**University of Birmingham**	23	24	10	58%	16 (5-19)	30%	19 (13-19)	18%	19 (13-20)
**King's College London**	27	40	18	55%	17 (9-20)	48%	13 (6-17)	26%	15 (8-18)
**Cardiff University**	18	22	10	55%	18 (7-20)	56%	7.5 (4-16)	30%	14 (5-18)
**Queen's University Belfast**	7	8	4	50%	19.5 (4-20)	86%	2 (1-7)	43%	6 (1-18)
**Newcastle University**	20	22	11	50%	19.5 (9-20)	45%	15 (6-18.5)	23%	17 (9-19)

SI10. Caveats and justifications

The overall number of caveats for advice, causation (main and secondary statements) and human inference was low in PRs (14%, 11%, 4%) and news stories (16%, 14%, 0%), and was strongly associated between news and PR: of the news with caveats, 57% and 50% had PRs with caveats. This can be compared to simulated levels of chance association of 1.3% (± 0.9% 95% CI) and 2.2% (±0.7% 95% CI), derived similarly to the simulations described in SI7, above. Arguably, such context is always relevant, to draw attention to the research being correlational or based on animal research, and to allow readers to distinguish, for example, between a simple correlation on a small sample and a large study that controlled for many other factors. The frequency of caveats we found was even lower than that reported by Woloshin et al. (2009; ref 20 in main text) in PRs issued by 20 academic medical centres,

where 42% did not provide any relevant caveats and 90% about animal or laboratory studies lacked caveats about extrapolating to humans.

Caveats for advice were not significantly associated with news uptake: 7/12 PRs with caveats had news (58%); 46/78 PRs without caveats had news (59%); confidence intervals on the difference are -29% to +32%; $\chi^2(1)=0.002$, p=0.97. Caveats for causation were possibly associated with increased news uptake from 94/190 (49%) for PR without caveats to 17/24 (71%) for PR with caveats ($\chi^2(1)=3.9$, p=0.05 uncorrected; bootstrapped 95% confidence intervals of the difference are 1% to 40%). Caveats for human inference were too few to further analyse. Overall, Ns for caveats are too small for strong conclusions about news uptake.

Similarly, the number of justifications for advice, causal claims and human inference was low both in PR (13%, 14%, 11%) and in news (17%, 10%, 3%), and strongly associated between news and PR: of the news with justifications, 45%, 87% and 50% had PRs with justifications compared to simulated chance associations of 1.2% (± 0.8%), 1.5% (± 0.6%) and <1%. There were no significant associations between presence/absence of justifications and news uptake, though Ns for justifications are too small for strong conclusions. For advice 7/11 [64%] with justifications had news, 46/79 [58%] without justifications had news, CIs of the difference are -27% to +26%; $\chi^2(1)=0.1$, p=0.7. For causation 11/25 [44%] with justifications had news, 97/177 [55%] without justifications had news, CIs of the difference are -11% to +32%; $\chi^2(1)=1.0$, p=0.3. For human inference 1/5 with justifications had news, 21/41 without justifications had news, CIs of the difference are -20% to +63%; small N violates χ^2 assumptions.

SI11. Indicators of news sources.

To estimate the relative importance of PR as the main source for the science stories in our sample, independently from the factors analysed for our main questions, we used dates of release, quotes and study details. Note that these estimates do not necessarily reflect all science news, given that the news stories in our sample were purposely selected to be on the same studies as those in our PR materials.

Dates. For selecting news stories, we used a criterion of release date being within 30 days of the PR. In fact, 580/668 (87%) of news stories were released within a day of the PR release date.

Quotes. We coded up to four quoted sources in news stories. Of the 668 analyzed news stories, 592 (89%) had quotes; 427 (72%) of these stories contained quotes identical to those included in the PR; 263 (44%) had alternative or additional quotes from the authors of the associated peer-reviewed journal article; 29 (5%) contained quotes identical to text in the journal article; 50 (8%) had quotes from other sources (e.g. funders) related to the research; and 179 (30%) had quotes from independent scientists or 'experts'.

Study details. We coded whether and how accurately/precisely each PR and news story reported sample size (N), completion rate, length of study, and number of time points for longitudinal studies. Of these data, we asked how often news stories provide details that were not contained in the associated PR (i.e. as evidence that the journalist has used a source additional to the PR). There were 89 news stories for which the PR did not give any indication of N where N could have been reported. Of these, only 2 stories reported N exactly, 7 reported N approximately (within 20%), and 6 reported N with more than 20% discrepancy with the actual N. Similarly, only 5% (16) news stories mentioned completion rate out of the 346 news stories where it was relevant but the PR did not provide it. Likewise, only 3% (9) of 280 news stories gave study duration where PR did not and only 6% (2/36) gave the number of time points in longitudinal studies where the PR did not.

For comparison, when PRs do provide these numbers, journalists often use them. N was reported exactly in the news in 57% of cases (89/157), and approximately in a further 13% of stories. Completion rate was reported in 53% (67/127) of news, while duration was reported in 67% (118/176). Similarly, of 81 PRs that mentioned time points for longitudinal studies, 57 news stories mentioned them as well (70%).

SI12. Scientist doublethink

Whilst instigating the main study, we performed an online survey of scientists' attitudes toward science in the media, and their experiences with PR. We advertised the survey via the *Guardian*, the BBSRC, and social media. The sample is self-selected and likely biased towards pre-existing interest in the topic of science news and by the subject area distribution in our advertising routes. As expected, the respondents (N=248) blamed journalists more than any other party for misreporting in science news. However, 79% of scientists who had PRs about their work reported involvement with those PRs, and despite this involvement, 32% acknowledged that their PRs were exaggerated (Figure S2). Thus it appears that some scientists do have awareness that PRs are a source of misreporting, but as a group we appear to engage in doublethink - colluding in producing exaggerated PRs but mainly blaming the media for the shortcomings of science news.

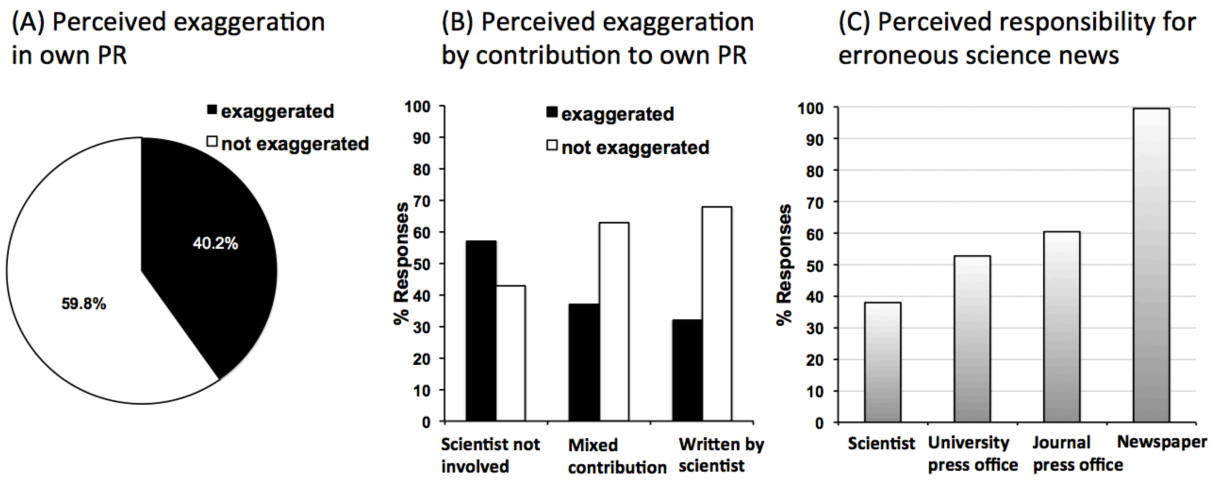

Figure S2. We performed an online survey in March 2012 to gather a sample of scientist's experiences and opinions on PR and science news (N=248). The key results were that 40% (N=43) of respondents with experience of PR (N=107) perceived that their most recent PR was exaggerated (A). Unsurprisingly, this proportion decreased with greater levels of declared involvement in the preparation of PRs, but still remained above 30% even for those scientists who reportedly wrote the PR themselves (B). When asked who was responsible for erroneous science news (C), 30-60% attributed some responsibility to scientists and press offices; this may reflect awareness of some PR exaggeration. However, 100% of respondents attributed responsibility to newspapers. The survey and accompanying data can be downloaded from http://dx.doi.org/10.6084/m9.figshare.903704. We advertised the survey via the *Guardian*, the BBSRC, and social media. The sample is self-selected (likely due to pre-existing interest in the topic of science news) and possibly biased by the subject area distribution in our advertising routes.

SI13. Comparison between news outlets and journalist type

Table S5 shows the rates of inflation from PR to news and from JA to news for different outlets. As in Table S4, advice, causal claims and human inference are combined to form one category of 'statements', which is why N statements differs from N PR. Note that the percent of statements *without* inflation is given (rather than *with* inflation). Some news outlets had too few N to be included individually: The Mail on Sunday (N=1) and Mail Online (N=2) have been combined with The Daily Mail (N=89), The Sunday Sun (N=1) has been combined with The Sun (N=44), The Sunday Telegraph (N=1) has been combined with The Telegraph (N=80), The Sunday Times (N=1) has been combined with The Times (N=31); The Daily Star, The Economist, the New Scientist, and the Press Association (each N<6) have been excluded. Note that the reason N statements differ slightly between the comparisons to PR and to JA is because in some cases comparison could not be made, for example if the PR does not say anything upon which to base a code for animal vs human, then human/animal claims in news could not be compared to PR.

The table is ordered by % claims without inflation from PR to news, though we do not make any conclusions from this order, given the very wide confidence intervals on the ranks (calculated as for Table S4). The more appropriate conclusion appears to be that news outlets do not differ from each other as much as might be generally assumed.

We also coded whether the journalist for each news story was a generalist or health/science specialist. Counter to expectation, we detected no differences between these categories for inflation rates. For advice, there were 23 inflations from PR in 182 news stories for specialists (13%) compared to 21/179 (12%) for generalists (difference =0.9%, with 95% CI of -5.8% to 7.6%). For causal claims from correlational results, there were 71/201 (35%) for specialists vs 83/244 (34%) for generalists (difference =1.3%, with 95% CI of -7.7% to 10.1%). For human inference from non-human studies, there were 5/57 (9%) for specialists vs 12/95 (13%) for generalists (difference =-3.9%, with 95% CI of -13.7% to 6.7%). It may be noteworthy that specialists wrote about non-human studies less frequently than did generalists, which may indicate differing knowledge about the difficulties of translating animal results into treatments for humans.

Table S5. Rates of inflation for news outlets in our study. Inflation is listed relative to PRs and relative to JAs (see text above for further explanation).

	N stories	N statements	N inflated PR to news	% without inflation	Rank (95% CI)	N statements	N inflated JA to news	% without inflation	Rank (95% CI)
**The Mirror**	50	47	5	89%	1 (1-7)	52	14	73%	1.5 (1-8)
**The Independent**	40	55	8	85%	2 (1-9)	53	21	60%	8 (2-12)
**The Scotsman**	23	32	5	84%	3 (1-11)	32	11	66%	5 (1-12)
**BBC news online**	95	120	20	83%	4.5 (1-9)	120	46	62%	7 (3-11)
**The Express**	57	66	11	83%	4.5 (1-10)	67	32	52%	12 (6-12)
**The Times**	32	35	6	83%	6 (1-11)	36	12	67%	4 (1-11)
**Reuters**	49	68	13	81%	7 (2-11)	66	21	68%	3 (1-9)
**Metro**	49	56	11	80%	8 (1-11.5)	52	14	73%	1.5 (1-7)
**The Telegraph**	81	110	28	75%	9 (5-12)	112	39	65%	6 (2-10)
**The Guardian**	44	65	17	74%	10 (4-12)	67	28	58%	9 (3-12)
**The Sun**	45	48	13	73%	11 (4-12)	48	22	54%	10 (4-12)
**The Daily Mail**	92	118	34	71%	12 (7-12)	118	55	53%	11 (6-12)
